# Assessing risk factors for elevated creatine kinase levels as an indicator of compartment syndrome following laparoscopic or robot-assisted colorectal cancer surgery in the lithotomy-trendelenburg position

**DOI:** 10.1007/s00464-024-11209-8

**Published:** 2024-08-30

**Authors:** Chikako Kusunoki, Mamoru Uemura, Mitsunobu Takeda, Yuki Sekido, Tsuyoshi Hata, Atsushi Hamabe, Takayuki Ogino, Norikatsu Miyoshi, Yoshinori Kagawa, Mitsuyoshi Tei, Hidetoshi Eguchi, Yuichiro Doki

**Affiliations:** 1 Department of Gastroenterological Surgery, Graduate School of Medicine, Osaka University, 2-2 Yamada-Oka, Suita City, Osaka, 565-0871 Japan; 2 Department of Gastroenterological Surgery, Osaka International Cancer Institute, Osaka, Japan; 3 Department of Surgery, Osaka Rosai Hospital, Sakai, Japan

**Keywords:** Well leg compartment syndrome (WLCS), Lithotomy position, Colorectal surgery, Creatine kinase (CK)

## Abstract

**Background:**

Well-leg compartment syndrome (WLCS) can occur due to compression and lower limb circulation disturbances caused by the surgical position during the procedure. Although rare, with an incidence of 1 in 3500 surgeries performed in the lithotomy position, it can lead to serious complications. Therefore, prevention and early diagnosis are critical. Symptoms of WLCS, such as leg pain, swelling, paresthesia, and serum creatine kinase (CK) levels are useful for diagnosis. This study aimed to investigate the risk factors for postoperative CK elevation in laparoscopic or robot-assisted colorectal cancer surgery performed in the lithotomy-Trendelenburg position.

**Methods:**

Postoperative CK levels were measured in 178 patients who underwent laparoscopic or robot-assisted colorectal cancer surgery between February 2022 and March 2023. We compared patient backgrounds, short-term outcomes, and thigh/calf circumferences between patients with CK levels ≥ 250 (n = 62) and those with CK levels < 250 (n = 116). We investigated risk factors for elevated CK levels using both univariate and multivariate analyses.

**Results:**

Four patients with CK levels of 22405 U/L, 4685 U/L, 4050 U/L, and 3824 U/L reported symptoms, which improved with conservative treatment. The following independent prognostic factors were identified by multivariate analysis: male sex (odds ratio [OR], 4.403; 95% CI, 1.960 to 9.892), rectal surgery (OR, 2.779; 95% CI, 1.249 to 6.184), continuous head-down position duration ≥ 180 min (OR, 3.523; 95% CI, 1.552 to 7.997), and preoperative calf circumference ≥ 33 cm (OR, 2.482; 95% CI, 1.154 to 5.339).

**Conclusions:**

Risk factors for CK elevation after colorectal cancer surgery in the lithotomy position include male sex, rectal surgery, an extended continuous head-down position without position changes, and a larger preoperative calf circumference. This study highlights the potential importance of intraoperative position changes every 3 h for preventing elevated CK levels, although the preventive effect was not specifically examined.

The lithotomy position, where the patient is placed in the supine position with both legs elevated and open, knees bent, and lower legs secured for leg support, is commonly used in urological, gynecological, and gastrointestinal surgeries. The Trendelenburg position is commonly used in colorectal cancer surgery and involves positioning the body in a supine position with the legs elevated above the head. The lithotomy-Trendelenburg position can cause circulatory disorders in the lower legs due to the flexion of the hip and knee joints, as well as neuropathy due to nerve compression by boot-shaped leg supports. The circulatory disorder and compression of the lower legs can lead to ischemia and necrosis of muscle, resulting in muscle swelling and edema, which increases compartment pressure [1–3]. Well-leg compartment syndrome (WLCS) is a very rare complication that occurs in 1 in 3500 patients but is a serious complication that can lead to neuropathy, lower leg amputation, and acute renal failure due to rhabdomyolysis, requiring prevention, early diagnosis, and treatment [4, 5]. Serum creatine kinase (CK) elevation is a highly sensitive indicator of muscle damage and is useful in the diagnosis of WLCS [6].

Laparoscopic and robot-assisted colorectal cancer surgery has been increasingly recognized for its benefits in minimally invasive surgery. However, the extended surgical times required for advanced and recurrent cases have raised concerns about the increased risk of WLCS. Therefore, it is important to examine the risk factors for WLCS in laparoscopic and robot-assisted colorectal cancer surgery. The risk factors for WLCS have been reported, but no study has precisely analyzed these risk factors using CK, which is an objective quantitative indicator directly related to rhabdomyolysis. This paper aimed to investigate the risk factors for WLCS in detail using CK levels as an objective quantitative indicator and to provide rational evidence for the prevention of WLCS in laparoscopic and robot-assisted colorectal cancer surgery performed in the lithotomy-Trendelenburg position.

## Materials and methods

### Patients

We prospectively collected clinical data, including CK values and several factors related to colorectal cancer surgery, from 178 patients who underwent laparoscopic or robot-assisted surgery for colorectal cancer at our department between February 2022 and March 2023. The information reviewed included age, sex, body mass index (BMI), American Society of Anesthesiologists physical status (ASA-PS) classification, comorbidities, smoking history, blood test results, and medical history. We also reviewed short-term surgical outcomes, including the duration of anesthesia, the duration of surgery, pneumoperitoneum duration, continuous head-down position duration, blood loss volume, need for blood transfusion, use of compression stockings or intermittent pneumatic compression devices (IPCDs), and occurrence of venous thromboembolism (VTE). Serum CK levels were measured before surgery and on the day after surgery, with additional tests performed as needed. The thigh and calf circumferences were also measured at the maximum girth. The thigh circumference was calculated as the average of the maximum right and left circumferences. The calf circumference was calculated in the same way. Preoperative thigh and calf circumferences were measured upon patient admission to the hospital. Postoperative circumferences were measured the day after surgery, while the patient was wearing compression stockings, just before they got out of bed for the first time. No patients had conditions such as cerebral infarction or myocardial infarction that could increase serum CK levels.

### Statistical analysis

Numerical data are presented as medians with interquartile ranges (IQRs). Categorical variables were analyzed using Fisher’s exact test or the chi-square test, as appropriate. Differences in quantitative parameters were compared using the Wilcoxon signed-rank test. A *p*-value less than 0.05 was considered to indicate statistical significance. Univariate and multivariate analyses were performed to identify factors associated with the development of postoperative elevation of serum CK levels. All statistical analyses were carried out using JMP Pro software version 17.0.0 for Mac (SAS Institute Inc., Cary, NC, USA).

## Results

Patient characteristics are summarized in Table 1. A total of 178 patients underwent laparoscopic or robot-assisted colorectal cancer surgery from February 2022 to March 2023. Among these patients, 62 had postoperative day 1 serum CK levels of 250 U/L or higher (elevated CK group), and 116 had serum CK levels of 250 U/L or lower (normal CK group). The median age of the patients in the elevated CK group was significantly younger, and the group included more males than females. There were more current and past smokers in the elevated CK group because the highest percentage of tobacco users were male. There were more males than females in the elevated CK group. No significant differences were observed between the groups in terms of BMI or ASA-PS classification.

**Table 1:** Patient Demographics and Clinical Characteristics

	Total n = 178	CK ≥ 250 n = 62	CK < 250 n = 116	*p-value*
Age, median (IQR^1^)	70(61–77)	69(52.5–74.3)	71.5(64–78)	*0.0297*
Sex, [male/ female], n (%)	100(56.2)/78(43.8)	48(77.4)/14(22.6)	52(44.8)/64(55.1)	< *.0001*
BMI^2^, median (IQR)	22.4(19.7–24.9)	23.3(20.3–25.5)	22.1(19.6–24.2)	*0.0643*
ASA-PS^3^ ≥ 3, n (%)	17(9.5)	7(11.3)	10(8.6)	*0.5980*
Comorbidity, n (%)				
HT^4^, n (%)	51(28.7)	11(17.7)	40(34.5)	*0.0234*
DM^5^, n (%)	28(15.7)	8(12.9)	20(17.2)	*0.5216*
HL^6^, n (%)	24(13.6)	6(9.8)	18(15.7)	*0.3593*
Current & past smoker, n (%)	69(38.8)	35(56.5)	34(29.3)	*0.0006*
Preoperative				
CA19-9^7^ (U/mL)	13.8(8.3–30)	11.4(7.3–23.8)	14.8(9.5–39.2)	*0.0156*
CEA^8^ (ng/mL)	3(2–5.8)	3(2–6)	3(2–5)	*0.7740*
D-dimer (μg/mL)	0.6(0.3–1.3)	0.6(0.3–1.3)	0.64(0.3–1.4)	*0.5784*
Hb^9^ (g/dL)	12.5(10.9–13.4)	12.8(11.7–14)	12.2(10.6–13.2)	*0.0060*
Plt^10^ (× 10^4^/μL)	24.8(21.1–29.9)	23(20.6–26.7)	25.4(21.5–31)	*0.0288*
CK^11^ (U/L)	81(57–108)	88(70–124)	73(50–99.8)	*0.0031*

1. IQR; Interquartile range, 2. BMI; Body Mass Index, 3. ASA-PS; American Society of Anesthesiologists physical status, 4. HT; High blood pressure, 5. DM; Diabetes mellitus, 6. HL; Hyperlipemia, 7. CA19-9; Carbohydrate antigen 19-9, 8. Carcinoembryonic antigen, 9. Hb; Hemoglobin, 10; Platelet, 11; Creatine kinase

Table 2 details the short-term outcomes according to surgical procedure. The tumor location in the rectum was more common in the elevated CK group than in the normal CK group (74.2% vs 44%, p = 0.0001). The durations of anesthesia, operation, pneumoperitoneum, and continuous head-down position were longer in the elevated CK group compared to the normal CK group (anesthesia duration, median 489 vs 306.5 min, p < 0.0001; operation duration, median 417.5 vs 236 min, p < 0.0001; pneumoperitoneum duration, median 364 vs 193 min, p < 0.0001; head-down position duration, median 288 vs 162 min, p < 0.0001). The blood loss volume was significantly greater in the elevated CK group compared to normal CK group (median, 50 vs 20 ml, p < 0.0001). In almost all cases, 177 patients (99.4%) wore compression stockings, 172 patients (96.6%) used IPCD, with no significant differences between the two groups regarding the use of compression stockings and IPCDs. Regarding to the selection type of IPCD, the foot type was more common than the leg type in the elevated CK group (leg type/foot type: 60/40% vs 80/20%, p = 0.0068). No patients in the elevated CK group developed VTE.

**Table 2:** Short-term outcomes according to surgical procedure

	Total n = 178	CK^4^ ≥ 250 n = 62	CK < 250 n = 116	p-value
Tumor location [Rectum/Other], n (%)	97(54.5)/81(45.5)	46(74.2)/16(25.8)	51(44)/65(56)	*0.0001*
Anesthesia duration (min), median (IQR^1^)	347.5(275.8–469)	489(357.5–739.8)	306.5(251.5–367.3)	< *.0001*
Operation duration (min), median (IQR)	274(209–385.5)	417.5(285.5–638.5)	236(188–295)	< *.0001*
Pneumoperitoneum duration (min), median (IQR)	222(162.5–329)	364(233.5–566.5)	193(131.5–246.5)	< *.0001*
Continuous head-down position duration (min), median (IQR)	189(122–280)	288(187–443)	162(98–227)	< *.0001*
Blood loss (ml), median (IQR)	20(5–60)	50(20–100)	20(0–47.5)	< *.0001*
Blood transfusion + , n (%)	3(1.7)	1(98.4)	2(1.7)	*1.0000*
Compression stockings + , n (%)	177(99.4)	62(100)	115(99.1)	*1.0000*
IPCD^2^ + , n (%)	172(96.6)	60(96.8)	112(96.6)	*1.0000*
Leg type, n (%)/ Foot type, n (%)	128(73.1)/47(26.9)	36(60)/24(40)	92(80)/23(20)	*0.0068*
Postoperative VTE^3^ + , n (%)	1(0.6)	0(0)	1(0.9)	*1.0000*

1. IQR; Interquartile range, 2. IPCD; intermittent pneumatic compression devices, 3. VTE; venous thromboembolism, 4. CK; Creatine kinase

The patients’ thigh and calf circumferences are summarized in Table 3. Both the thigh and calf circumferences were significantly greater in the elevated CK group before and after the operation (preoperative thigh circumference: median 42.1 vs 41.3 cm, p = 0.0450; preoperative calf circumference: median 35 vs 32.7 cm, p = 0.0039; postoperative thigh circumference: median 42.5 vs 40 cm, p = 0.0074; postoperative calf circumference: median 33.8 vs 30.5 cm, p < 0.0001).

**Table 3:** The patient’s thigh and calf circumference

	Total n = 178	CK^3^ ≥ 250 n = 62	CK < 250 n = 116	p-value
Preoperative thigh circumference (cm), median (IQR^1^)	41.8(37.6–44.1)	42.1(37.4–45.8)	41.3(37.8–43.4)	*0.0450*
Preoperative calf circumference (cm), median (IQR)	33.3(31–36.5)	35(31.9–37.1)	32.7(31–35.8)	*0.0039*
Postoperative thigh circumference(POD^2^1) (cm), median (IQR)	40.5(37.7–43.8)	42.5(38.3–45.3)	40(37.4–42.5)	*0.0074*
Postoperative calf circumference(POD1) (cm), median (IQR)	31.5(29.3–34.3)	33.8(30.4–35.3)	30.5(29–33.5)	< *.0001*

1. IQR; Interquartile range, 2. POD; Post operative day, 3. CK; Creatine kinase

The risk of serum CK elevation was evaluated for each factor using univariate and multivariate analyses (Table 4). The cutoff values were set as follows: age, 70 years; continuous head-down position duration, 180 min; preoperative thigh circumference, 42 cm; and preoperative calf circumference, 33 cm, based on the median from our results. Univariate analysis revealed that male sex, a rectal tumor, a duration of continuous head-down position ≥ 180 min, a greater preoperative calf circumference ≥ 33 cm, and the use of an IPCD foot type were risk factors for CK elevation. Based on the results of the univariate analysis, factors with a p-value less than 0.05 were evaluated via multivariate analysis. Multivariate analysis revealed that male sex (odds ratio [OR]: 4.403; 95% confidence interval [CI]: 1.960–9.862, p = 0.0003), rectal cancer (OR: 2.779; 95% CI: 1.249–6.184, p = 0.0122), a longer continuous head-down position duration ≥ 180 min (OR: 3.523; 95% CI: 1.552–7.997, p = 0.0026), and a thicker preoperative calf circumference ≥ 33 cm (OR: 2.482; 95% CI: 1.154–5.339, p = 0.0200) were independent risk factors for postoperative CK elevation.

**Table 4:** Univariate and multivariate analyses of clinical characteristics associated with CK elevation

Variable	Univariate			Multivariate		
	OR	95%CI	*p-value*	OR	95%CI	*p-value*
Age (year-old) [< 70/ ≥ 70]	1.548	0.832–2.877	*0.1675*			
Sex [Male/Female]	4.220	2.098–8.487	< *.0001*	4.403	1.960–9.892	*0.0003*
Tumor location [Rectum/ Other]	3.664	1.862–7.209	*0.0002*	2.779	1.249–6.184	*0.0122*
Continuous head-down position duration (min) [≥ 180/ < 180]	5.534	2.707–11.312	< *.0001*	3.523	1.552–7.997	*0.0026*
Preoperative thigh circumference (cm) [≥ 42/ < 42]	1.294	0.657–2.547	*0.4562*			
Preoperative calf circumference (cm) [≥ 33/ < 33]	2.742	1.427–5.268	*0.0025*	2.482	1.154–5.339	*0.0200*
IPCD^1^ [ ±]	1.071	0.203–7.886	*0.9373*			
IPCD type [Foot type/Leg type]	2.667	1.338–5.314	*0.0053*	1.722	0.758–3.912	*0.1944*

1. IPCD; intermittent pneumatic compression devices

The details of the 14 patients who had a postoperative serum CK level of 1000 U/L or higher are shown in Table 5. Thirteen of the 14 patients were male. All patients underwent surgery for rectal tumors, and 8 patients underwent lateral lymph node dissection (LLND). Two patients underwent resection for locally recurrent rectal cancer (LRRC), and one patient underwent total pelvic exenteration (TPE). Patients who underwent these more invasive procedures tended to have a longer continuous head-down position duration and more blood loss. The thigh and calf circumferences of 11 patients was greater than the median of the overall patient population. Four patients reported lower leg pain, numbness, paresthesia, and edema at CK levels of 22405 U/L, 4685 U/L, 4050 U/L, and 3824U/L, respectively. These patients were examined by orthopedic surgeons and suspected of having WLCS. It was determined that the patient did not require a fasciotomy and could be monitored conservatively. Their symptoms and CK levels were regularly checked. Within 7–11 days postoperatively, CK levels returned to the normal range.

**Table 5:** The details of 14 patients who had a postoperative serum CK level of 1000 U/L or higher

Case	Age	Sex	BMI^1^	Approach	Tumor location	Operative procedure	Blood loss volume (ml)	Continuous head-down position duration (min)	Preoperative thigh circumference (cm)	Preoperative calf circumference (cm)	Postoperative CK^2^ (U/L) POD^3^1	Postoperative CK (U/L) maximum	Symptom
1	65	M	23.4	Robot	Rb	ISR^5^, LLND^6^	540	659	45.8	39	22405	22405	Lower leg edema, pain, Numbness
2	42	M	23.8	Robot	Rb-P	APR^7^, LLND	60	448	44.5	35	6070	6310	–
3	60	M	24.9	Lap	LRRC^4^	ISR, LLND sacrectomy	500	308	41.8	39.3	4050	4988	Leg paresthesia
4	43	F	18.8	Robot	Rb	LAR^8^, LLND	60	218	37.3	30.5	2410	2490	–
5	53	M	32.2	Robot	Ra	LAR	4900	176	53.5	42	1675	2450	–
6	85	M	25.7	Lap	Rb	APR	490	150	45.3	33.8	1347	1347	–
7	32	M	22.5	Robot	Rb	ISR	60	563	44.5	37.3	1158	1158	–
8	72	M	22.7	Robot	Ra-Rb	LAR, LLND	100	454	46.8	34.5	2122	2122	–
9	33	M	18.7	Lap	Rb	TPE^9^	60	471	37.5	29.8	4685	4685	Lower leg pain, Paresthesia, Numbness
10	67	M	36	Robot	P	APR, LLND	510	471	51.6	38.3	3824	3825	Leg pain
11	71	M	24.4	Robot	Rb	LAR(TaTME^10^), LLND	180	590	45.8	38.8	1108	1108	–
12	48	M	23.7	Robot	Rb	APR, LLND	55	346	45	36.5	1408	1408	–
13	69	M	22.6	Robot	LRRC	ISR	910	1098	41	36.5	1165	1165	–
14	61	M	26.7	Robot	Rb	LAR(TaTME)	50	175	43.5	36.3	1004	1004	–

1. BMI; Body Mass Index, 2. CK; Creatine kinase, 3. POD; Post operative day, 4. LRRC; Locally recurrent rectal cancer, 5. ISR; Intersphincteric resection, 6. Lateral lymph node dissection, 7. APR; Abdominoperineal resection, 8. LAR; Low anterior resection, 9. TPE; Total pelvic extenteration, 10. TaTME; Transanal total mesorectal excision

## Discussion

In this study, serum CK levels were measured the day after surgery to evaluate risk factors related to WLCS. Elevated serum CK is the most sensitive indicator of muscle injury, and it increases within 12 h of the onset of muscle injury. The upper limit of normal for serum CK is 250 U/L, with a serum CK over 1000 U/L indicating possible rhabdomyolysis and over 5000 U/L indicating a severe case of rhabdomyolysis [7]. The risk of elevated CK levels associated with the pathology of WLCS was examined to identify risk factors leading to WLCS. To evaluate potential WLCS risk, a cutoff of 250 U/L, which is the upper limit of normal CK, was used to compare the normal CK group with the elevated CK group. In this study, patients with CK levels of 3824 U/L, 4050 U/L, 4685 U/L, and 22,405 U/L, experienced symptoms such as pain in the lower legs. When CK levels exceed 5000 U/L, rhabdomyolysis is considered severe [7], and our study indicates that this generally correlates with the appearance of clinical symptoms.

Prolonged operative durations, pneumoperitoneum, and the head-down position are considered risk factors for WLCS. Pneumoperitoneum becomes a risk factor due to the reduction in venous return it causes [8]. In our study, patients with elevated CK levels had longer anesthesia durations, operative durations, pneumoperitoneum durations, and durations in the head-down position. A head-down position for more than 3 h and rectal surgery were also risk factors. Therefore, particular attention should be paid during laparoscopic and robot-assisted surgeries for advanced recurrent rectal cancer, which typically require long operative durations and tend to result in significant blood loss [9].

In colorectal cancer surgery, it is common to use both compression stockings and IPCDs for VTE prevention, but it is suggested that this increases pressure on the lower leg, potentially leading to an increase in WLCS. In this study, almost all patients wore compression stockings, and the rate of IPCD use was also high; however, the presence or absence of IPCDs did not affect CK elevation. Interestingly, in the CK elevation group, the rate of wearing IPCDs of the foot sole type was high. However, multivariate analysis did not identify it as an independent risk factor.

Young patients, particularly males, are considered to have an increased risk of WLCS, which is thought to be due to greater muscle mass [5, 8, 10]. In patients undergoing laparoscopic colorectal surgery in the lithotomy-Trendelenburg position, it has been reported that the preoperative maximum calf circumference correlates with the maximum external pressure in the calf region during surgery, and a larger maximum calf circumference can be a risk factor for WLCS [11]. In our study, male sex and a calf circumference of 33 cm or more were independent risk factors.

The compartment pressure in the lower legs is normally 0–8 mmHg, but it increases when the legs are placed in the lithotomy-Trendelenburg position, reaching an average of 30 mmHg after 5 h, which can have clinical implications. It has been reported that compartment pressure rapidly returns to less than 10 mmHg when the lithotomy position is released and the patient is placed in a supine position [12]. Furthermore, in patients undergoing laparoscopic surgery for colorectal cancer in the lithotomy-Trendelenburg position, the maximum external pressure in the calf region decreased when the patients were returned to a horizontal position for 5 mi [11]. Reports of WLCS in abdominal-pelvic surgery, including open surgery and surgeries other than colorectal cancer, suggest that operation time in the lithotomy position exceeding 4 h is a risk factor [8, 13–15]. There are no studies evaluating the risk of WLCS based on the continuous time spent in the lithotomy-Trendelenburg position during laparoscopic or robot-assisted colorectal cancer surgery. While there are studies that examine the duration of surgery, none specifically focus on the duration of the lithotomy-Trendelenburg position. In our study, continuous head-down positioning for more than 3 h was an independent prognostic factor. Considering the report that compartment pressure rapidly returns to normal, and the maximum external pressure in the calf region decreased for 5 min when the patient is placed in a supine position, this study highlights the potential importance of periodically returning the patient to a flat position for 5 min every 3 h to prevent elevated CK levels.

This study had several limitations. First, it was a single-center analysis with a limited number of cases. Second, since WLCS is a rare complication, we did not directly analyze the occurrence of WLCS as an event; instead, we examined the risk related to WLCS using CK levels as an objective indicator. For a more accurate risk assessment, a statistical analysis with an even larger sample size is needed. Additionally, further studies building on the results of this study are necessary to establish effective preventive measures against WLCS.

## Conclusion

Risk factors for CK elevation after laparoscopic or robot-assisted colorectal cancer surgery in the lithotomy position include male sex, rectal surgery, an extended continuous head-down position without changes for ≥ 3 h, and a larger preoperative calf circumference ≥ 33 cm. This study suggested the potential importance of intraoperative position changes every 3 h for at least 5 min for preventing elevated CK levels.

## Declarations

### Research involving human and animal participants

N/A.
